# Exploring wave propagation dynamics in heterogeneous excitable media: effects of intermittent cellular inactivation

**DOI:** 10.3389/fphys.2026.1900002

**Published:** 2026-07-23

**Authors:** Yaacov Biton, Doron Braunstein, Ella Smolik, Revital Rabinovitch, Avinoam Rabinovitch

**Affiliations:** 1 Department of Physics, Ben-Gurion University of the Negev, Be'er, Sheva, Israel; 2 Department of Physics, Shamoon College of Engineering, Be'er, Sheva, Israel; 3 Makif Yud Alef High School, Rishon LeZion, Israel

**Keywords:** excitable media, FitzHugh-Nagumo model, intermittent cellular inactivation, functional heterogeneity, wave propagation, spiral waves, atrial fibrillation

## Abstract

Spatially and temporally varying excitability is increasingly recognized as an important source of conduction instability in excitable media. In this study, we investigate the effects of localized intermittent cellular inactivation on wave propagation using a two-dimensional continuous FitzHugh-Nagumo reaction-diffusion model. A designated region of the medium was subjected to stochastic switching between excitable and weakly excitable states, thereby representing dynamic functional heterogeneity in otherwise homogeneous tissue. Systematic simulations revealed three distinct propagation regimes as a function of the density of intermittently inactive cells within the affected region. At low densities (<4%), planar waves traversed the heterogeneous zone with only minor local distortions and recovered global coherence. At intermediate densities (approximately 4%-18%), localized wave breaks promoted the initiation of persistent spiral activity. At higher densities (>18%), conduction through the heterogeneous region was severely disrupted, leading to wavefront fragmentation and failure of coherent propagation. These results show that temporally fluctuating local inactivation, even in the absence of fixed anatomical obstacles or deterministic ectopic drivers, is sufficient in this model to generate threshold-dependent transitions from stable propagation to reentrant-like dynamics. The findings support the idea that dynamic functional heterogeneity may provide a plausible mechanistic substrate for arrhythmogenic wave instability in cardiac-like excitable media, such as atrial fibrillation (AF), and motivate further investigation in more physiologically detailed models.

## 1 Introduction

Normal cardiac activation relies on a hierarchically organized conduction system, originating in the sinoatrial node and propagating through the atrioventricular node, His-Purkinje system, and ventricular myocardium (Keener and Sneyd, 2009). Disruption of this orderly sequence, whether by ectopic foci, reentrant loops, or wave break, constitutes the canonical framework of arrhythmogenesis (Haïssaguerre et al., 2016b). Cardiac intermittently inactive cells, i.e. cells that exhibit irregular functional activity can appear in two forms: active and passive. While the active type of intermittency has recently been abundantly investigated, such cells identified as ectopic drivers, namely irregular sources of electrical pulses (see, e.g., Haïssaguerre et al., 2016a; Gibo et al., 2025), the passive intermittent ones, that can either be activated by neighboring cells or fail to respond to their input, remains comparatively underexplored. In our recent paper (Rabinovitch et al, 2023), using a cellular automata model, we have shown that such intermittent passive cells are possibly one of the most powerful sources of atrial fibrillation.

In cardiac electrophysiology, intermittent behavior has traditionally been described in terms of electrophysiological remodeling rather than “intermittently inactive cells” per se. Regions exhibiting irregular activation, conduction delay, or transient block have been linked to ischemia, fibrosis, metabolic stress, and ion-channel remodeling, which collectively create substrates for atrial and ventricular arrhythmias, including atrial fibrillation (AF) and ventricular fibrillation (VF) (Iwamiya et al., 2024). Importantly, experimental and computational studies suggest that functional heterogeneity can emerge even in structurally intact tissue, through dynamic modulation of gap-junction conductance and ion-channel availability, producing transient, spatially distributed conduction instability (Attuel et al., 2019).

Simplified mathematical models of excitable media have demonstrated that spatial and temporal heterogeneity, even in the absence of anatomical obstacles, is sufficient to produce spiral waves, wave-break, and turbulence-like electrical dynamics. Classical implementations of the FitzHugh-Nagumo (FHN) reaction diffusion framework and related reduced-order models have reproduced key qualitative features of fibrillation, including filament instability and spiral wave breakup (Fenton and Karma, 1998). More recent work has shown that inhomogeneities in conduction anisotropy or dynamically modulated coupling alone can attract, repel, anchor, or destabilize spiral activity, giving rise to complex reentrant dynamics (Kuklik et al., 2013, 2012). Beyond the specific context of cardiac electrophysiology, considerable attention in the broader nonlinear dynamics literature has been devoted to understanding how noise and spatial heterogeneity influence wave propagation in excitable media. Notably, Perc (2005) demonstrated that noise can induce spatial coherence resonance, whereby stochastic fluctuations organize rather than disrupt spatial excitation patterns. Subsequent work by Gosak et al. (2009) further showed that the interplay between external pacemaking activity and noise can generate structured spatial periodicity. These studies highlight the constructive role of stochasticity in spatially extended excitable systems. Furthermore, the interaction between propagating wavefronts and obstacles has been recognized as a fundamental mechanism for the initiation of reentry; for instance, Rostami et al. (2018) systematically investigated how wavefront-obstacle interactions can lead to wave break and spiral wave formation.

However, while discrete models like cellular automata (Rabinovitch et al, 2023) provide valuable foundational insights into arrhythmogenesis, they are limited in their ability to capture continuous spatial gradients, wavefront curvature, and the nuanced temporal dynamics of repolarization. To bridge this gap, the present work extends this theoretical foundation by explicitly introducing temporally stochastic excitability into a continuous, reduced-order FitzHugh-Nagumo framework to model intermittent cellular inactivation. Building upon the aforementioned nonlinear dynamics literature, our approach introduces a critical distinction: rather than considering additive noise acting uniformly across the medium or static structural barriers, we focus on localized, temporally intermittent inactivation. In our model, this intermittently inactive region acts as a dynamic functional obstacle, whose spatial configuration changes stochastically over time, fundamentally altering wave-obstacle dynamics. In contrast to prior deterministic heterogeneity models and discrete frameworks, we implement these stochastic, spatially localized fluctuations in a 2D continuous medium to mathematically emulate intermittent conduction failure at the cellular and tissue levels.

By systematically varying the density of intermittently inactive cells within a spatially confined region, while keeping the switching probability and temporal update rule fixed, we demonstrate that dynamic functional heterogeneity can generate a spectrum of arrhythmia-like wave dynamics of these regions containing intermittently inactive cells, we demonstrate that functional heterogeneity alone in the absence of fixed anatomical obstacles, fibrosis, or autonomic triggers, is sufficient to generate a full spectrum of arrhythmic wave dynamics, ranging from benign conduction slowing to sustained spiral-wave turbulence. Crucially, our continuous model allows us to estimate model-dependent transition densities that separate distinct macroscopic propagation regimes under the present parameterization.

These findings provide a mechanistic bridge between the concept of passive cell intermittency and cardiac arrhythmogenesis, suggesting that stochastic modulations of excitability may constitute an independent and clinically relevant arrhythmogenic substrate. Furthermore, this framework offers a computationally efficient platform for exploring novel therapeutic strategies aimed at stabilizing excitable media by suppressing or controlling intermittent functional heterogeneity.

## 2 The model

Computational modeling has become an essential pillar of contemporary cardiac electrophysiology, enabling the systematic investigation of complex electrical phenomena that cannot be fully resolved through experimental approaches alone. Biophysically detailed frameworks such as the Rogers-McCulloch and Fenton-Karma models have been extensively employed to reproduce tissue-level electrical behavior with high physiological fidelity, incorporating the nonlinear interactions of individual cardiomyocytes and their emergent collective dynamics (Biasi and Tognetti, 2021; Fenton et al., 2002). These models typically rely on large sets of ionic current equations describing the kinetics of sodium, potassium, and calcium channels, thereby providing a granular representation of action potential morphology and conduction (ten Tusscher et al., 2004; Rudy and Silva, 2006).

However, when the scientific objective shifts from reproducing detailed ionic mechanisms to interrogating macroscopic nonlinear dynamics and emergent spatiotemporal patterns, reduced-order models become advantageous due to their computational tractability and conceptual transparency. Among these, the FitzHugh-Nagumo (FHN) system occupies a central role as a canonical representation of excitable media, distilling the biophysical complexity of Hodgkin-Huxley-type formulations into a two-variable dynamical system that preserves the essential qualitative features of excitability, refractoriness, and recovery (FitzHugh, 1955; Nagumo et al., 1962). The FHN framework has been widely adopted in cardiac modeling to study wave propagation, spiral wave formation, and arrhythmogenic dynamics, particularly in regimes where global pattern formation is the primary object of investigation (Fenton and Karma, 1998; Gray and Jalife, 1998; Yang et al., 2024).

Recent work has drawn attention to previously underappreciated arrhythmogenic mechanisms that cannot be adequately captured by purely deterministic or structurally static models. Rabinovitch et al. (2023) employed a cellular automaton framework to describe a novel, functionally defined arrhythmogenic substrate characterized by intermittent cardiomyocytes. In this paradigm, spatially confined regions exhibit stochastic transitions between excitable, refractory, and functionally inexcitable states, such that incoming wavefronts may be unpredictably transmitted, delayed, or blocked. This dichotomous and temporally fluctuating behavior introduces a dynamic form of electrical heterogeneity that is capable, under appropriate conditions, of destabilizing coherent wave propagation and giving rise to chaotic atrial activation consistent with atrial fibrillation (AF) (Rabinovitch et al., 2023). Importantly, such intermittent zones may also act as powerful arrhythmogenic amplifiers when combined with established structural substrates, including atrial fibrosis, altered gap-junction coupling, and prolonged action potential duration, all of which exacerbate conduction instability (Elliott et al., 2023; Nattel, 2017; Spach and Dolber, 1986).

Motivated by these observations, the present study adopts an extended FitzHugh-Nagumo framework designed to capture the interaction between classical excitable-media dynamics and stochastically fluctuating cellular excitability. The FHN system, originally derived as a phenomenological reduction of the Hodgkin-Huxley equations, reproduces the biphasic structure of the cardiac action potential through a fast activator variable governing rapid depolarization and a slow recovery variable modeling repolarization and refractoriness (FitzHugh, 1955). By explicitly incorporating intermittent cellular activation into this framework, we simulate the electrical consequences of spatially heterogeneous tissue in which subsets of cells undergo random transitions between excitable and functionally silent states. This extension enables a systematic assessment of how stochastic excitability modulates wavefront stability, conduction velocity, and susceptibility to reentrant activity, thereby complementing previous deterministic studies of pattern formation in excitable media (Fenton and Karma, 1998; Gray and Jalife, 1998.

Governing Equations: The nonlinear reaction-diffusion system employed in this study is defined by the following partial differential equations (**Equation 1**):

(1) $$\begin{array}{l} \frac{dv}{dt}=\text{D}\nabla^{2}v+v\left(v-a\right)\left(1-v\right)-w+I\left(t\right)\\ \frac{dw}{dt}=\epsilon \left(v-dw\right) \end{array}$$

Here, *v* denotes the dimensionless transmembrane potential, representing the local electrical activation state of the tissue, whereas *w* denotes a recovery variable that phenomenologically captures inhibitory and refractory processes following excitation. The diffusion coefficient *D* governs the spatial spread of electrical activity through nearest-neighbor coupling, representing effective intercellular conductivity at the tissue scale (Keener and Sneyd, 2009). Parameter values *ϵ* = 0.01, *d* = 3 and *D* = 1 were selected to approximate cardiac-like recovery kinetics, producing refractory periods and restitution behavior consistent with empirical observations (Nash and Panfilov, 2004; DeTalà et al., 2022).

The excitability control parameter, *a*, plays a central physiological role in this model. Negative values of *a* induce self-sustained oscillatory behavior and are therefore excluded from the present study. In the FitzHugh-Nagumo equations, *a* mathematically dictates the roots of the cubic nullcline, thereby determining the threshold for excitation. Smaller positive values (e.g., *a* = 0.12, designated “ready” cells) correspond to an excitable regime with a sufficiently low threshold, allowing an incoming activation wavefront to elicit a full regenerative action potential. As a increases, the local maximum of the cubic nullcline shifts, which progressively raises the required excitation threshold. Beyond a critical parameter value (approximately *a* ≈ 0.3 under the present parameterization), the system undergoes a transition where the resting state becomes globally stable; consequently, the cell loses its ability to fire a regenerative action potential entirely. We therefore use a high-a, supra-critical value, *a* = 0.54, to explicitly represent functionally “inactive” cells that reduce excitability and increase the likelihood of conduction slowing or functional block (Rudy and Silva, 2006; Keener and Sneyd, 2009). For an action potential to be elicited, the total input *I*(*t*) must be strong enough to drive the fast transmembrane potential *v* beyond this dynamic, *a*-dependent local maximum.

Specifically, each designated intermittent cell switches independently between the excitable state *a* = 0.12 and the inactive state *a* = 0.54 with a probability of *p* = 0.5 at each simulation time step Δ*t* = 0.25. This switching process lacks temporal persistence, functioning as a memoryless Markovian process. Consequently, the mean residence time in either state is intrinsically tied to the numerical integration step, yielding an average duration of 0.5 time units. Furthermore, the state transitions of neighboring intermittent cells are statistically independent, meaning there is no spatial correlation. We note that the switching timescale relative to the integration step, as well as the potential introduction of temporal or spatial correlations, represent highly relevant control parameters whose effects on wave propagation could be systematically explored in future work. The density of these intermittent cells within the heterogeneous rectangular region is fixed at *ρ*, while all cells outside this region remain permanently in the excitable state *a* = 0.12.

The external input term *I*(*t*) represents an injected current used to mimic incoming activation wavefronts. When *I*(*t*) exceeds the effective threshold determined by *a*, a full action potential is elicited; otherwise, the system remains in a resting but excitable state. The tissue is represented as a two-dimensional lattice of 800 × 800 grid points, discretized using uniform dimensionless spatial steps of Δ*x* = Δ*y* = 0.01. The computational domain is constrained by no-flux (Neumann) boundary conditions $\left(\frac{\partial v}{\partial n}\right)=0$, $\left(\frac{\partial w}{\partial n}\right)=0$, ensuring that wave propagation terminates at the tissue borders without artificial reflection or periodic wrap-around. A temporal integration step of Δ*t* = 0.25-time units was chosen to ensure numerical stability and adequate temporal resolution of wave dynamics (Keener and Sneyd, 2009).

Each grid point is assigned an excitability class according to its *a* value: ready cells (*a* = 0.12) are fully excitable, whereas inactive cells (*a* = 0.54) are functionally unresponsive. A spatially confined subset of grid points is designated as intermittently inactive cells, in which the parameter *a* undergoes stochastic switching between these two values over time. This stochastic modulation is implemented within a fixed rectangular region of interest, where a randomly determined proportion of nodes transitions between excitable and inexcitable states at each simulation step. A rectangular geometry was selected as a canonical representation of a localized pathological patch. While the specific outline of this region, such as sharp boundaries or corners, may locally influence wave anchoring and individual spiral trajectories, the present study focused on the effect of the density ρ of intermittently inactive cells within a heterogeneous region of fixed size. Thus, our robustness statement refers to the qualitative emergence of the three propagation regimes under this fixed localized-patch configuration, rather than to invariance with respect to arbitrary region dimensions. Changes in the size or aspect ratio of the intermittently inactive region would be expected to alter the effective interaction length between the propagating wavefront and the heterogeneous substrate and may therefore shift the numerical transition densities. This approach operationalizes the concept of dynamic, non-deterministic functional heterogeneity previously proposed as a driver of arrhythmogenesis (Rabinovitch et al., 2023).

Simulation Protocol: The coupled reaction-diffusion equations are solved numerically in MATLAB utilizing the Forward Euler method over the two-dimensional lattice, with each node interacting locally with its nearest neighbors to enable wave propagation. A planar wavefront is initiated at *t* = 0, propagating parallel to the Y axis of the grid. The simulation geometry is designed such that the left portion of the wave bypasses the intermittent region, while the right portion traverses it, creating a controlled comparison between homogeneous and heterogeneously modulated conduction. This configuration enables a systematic analysis of wavefront distortion (Fenton and Karma, 1998), conduction delay, fragmentation, and the emergence of reentrant activity induced by intermittent inactivation.

Through this framework, the model provides a computationally efficient and physiologically interpretable platform for dissecting the mechanistic role of dynamically fluctuating cellular excitability in generating electrical instability. The results offer insight into how passive intermittent functional heterogeneity, even in the absence of fixed structural lesions or fibrosis, can act as an autonomous arrhythmogenic substrate capable of initiating and sustaining complex patterns characteristic of fibrillation.

## 3 Results

**Figure 1:**
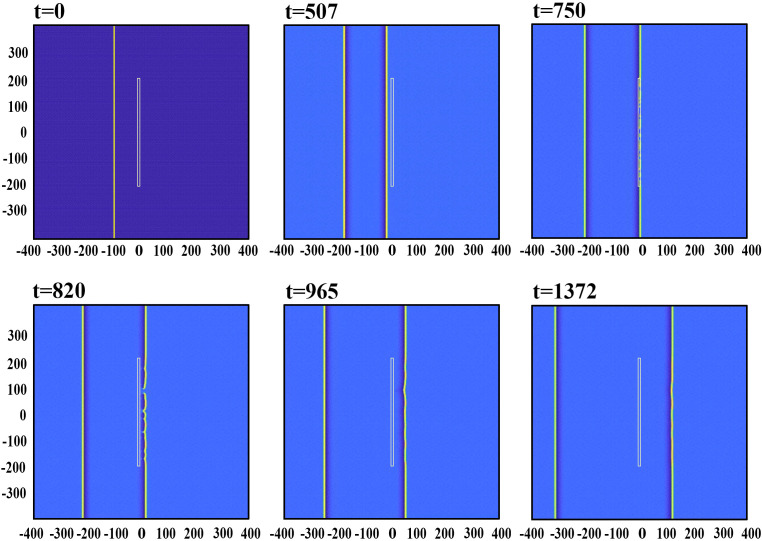
Uninterrupted planar wave propagation through a region containing a low density (<4%) of intermittently inactive cells. Initial wavefront at t = 0. Minor local distortions are observed within the heterogeneous zone (dashed rectangle: -7<X<0 and |Y|<200), yet the wave rapidly restores planar coherence. See Supplementary Movie at https://physics.bgu.ac.il/~yaacov/File/Scenario1.mp4 for the complete spatiotemporal evolution.

**Figure 2:**
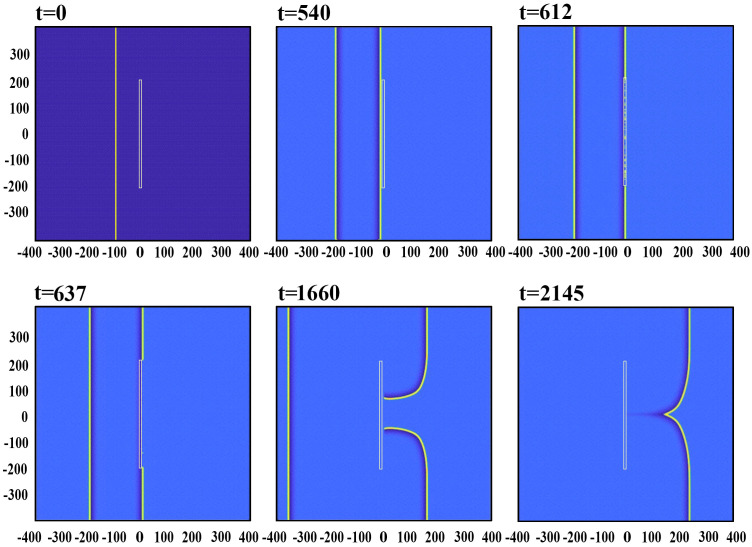
Wavefront fragmentation induced by a high density (>18%) of intermittently inactive cells. The advancing front is severed upon entering the heterogeneous region (dashed rectangle: -7<X<0 and |Y|<200), generating two free ends that curl and migrate. Partial reconnection occurs in some instances, but persistent distortion remains. See Supplementary Movie at https://physics.bgu.ac.il/~yaacov/File/Scenario2.mp4 for the complete spatiotemporal evolution.

**Figure 3:**
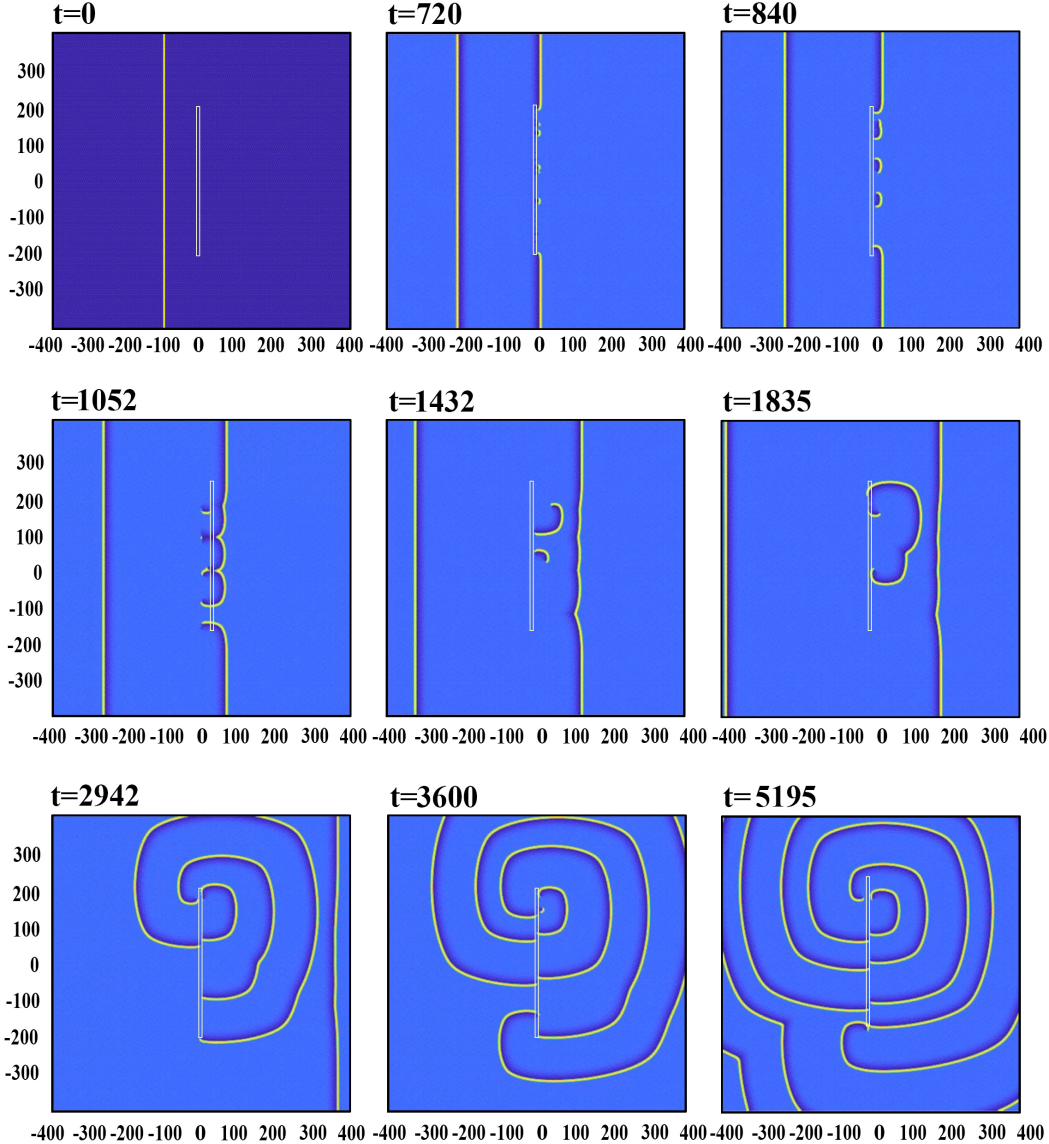
Emergence and persistence of spiral waves in the intermediate regime (4- 18% intermittently inactive cells). Multiple random wave-breaks within the heterogeneous zone (dashed rectangle) generate fragments of sufficient curvature to initiate ectopic self-sustaining spiral waves visible after ~720 time units. See Supplementary Movie at https://physics.bgu.ac.il/~yaacov/File/Scenario3.mp4 for the complete spatiotemporal evolution.

Numerical simulations were performed to examine how a spatially confined population of intermittently inactive cells affects the propagation of an initially planar excitation wave. The intermittently inactive cells were distributed within a rectangular heterogeneous region defined by -7< X< 0 and |Y|< 200, while the wavefront propagated from left to right across the tissue. Representative numerical examples are presented in **Figures 1**–**3**. These examples reveal three qualitatively distinct modes of wave propagation, which are subsequently quantified in Section 3.1 using the normalized wavefront length.

In the low-density regime (< 4% intermittently inactive cells), the propagating planar wavefront was only weakly affected by the heterogeneous region. As shown in **Figure 1**, the wavefront developed minor localized distortions while crossing the intermittently inactive region, particularly around t = 750 TU. However, these perturbations remained spatially limited, and after traversing the heterogeneous zone the wavefront largely recovered its planar geometry and preserved functional continuity. This mode of propagation is therefore referred to as Scenario 1: Uninterrupted Planar Propagation.

A markedly different response was observed at high densities of intermittently inactive cells (> 18%). In this regime, the heterogeneous region acted as a strong functional obstacle. Upon entering the region, the incoming planar wavefront was extensively disrupted and divided into disconnected propagating segments with free ends, as illustrated in **Figure 2**. The original planar morphology was therefore lost, and the excitation wave propagated as separated fragmented fronts. This mode is denoted as Scenario 2: Wavefront Fragmentation.

In the intermediate-density regime, approximately 4-18% intermittently inactive cells, the interaction between the planar wavefront and the heterogeneous region produced sustained reentrant dynamics. As shown in **Figure 3**, localized wave breaks generated within the heterogeneous region evolved into rotating spiral waves. These spirals persisted long after the initial planar wavefront had passed, indicating the emergence of self-sustained ectopic activity. This behavior is referred to as Scenario 3: Emergence of Persistent Spiral Waves.

**Figure 4:**
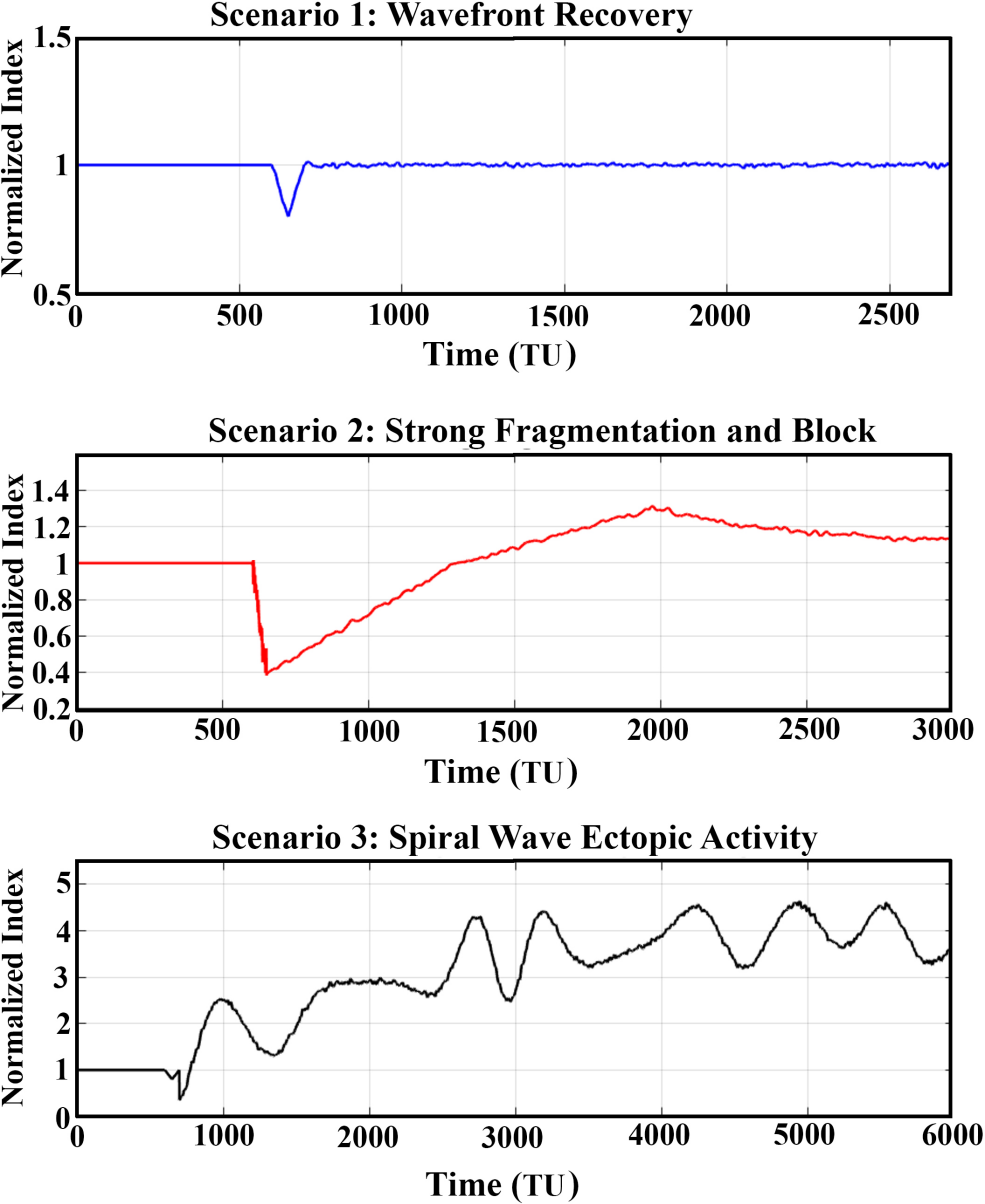
Temporal evolution of the Normalized Wavefront Length across the three dynamic propagation regimes. The index is normalized against the constant length of the undisturbed planar wave prior to its interaction with the heterogeneous region (t< 600 TU, baseline = 1.0). Top (Scenario 1): In the low-density regime (<4% intermittently inactive cells), the wavefront experiences a minor transient truncation before rapidly healing and recovering full spatial coherence. Middle (Scenario 2): At high densities (>18%), the wavefront is severely blocked and severed, causing an abrupt initial drop. Subsequent curling of the free ends temporarily increases the total active length before collision and partial reattachment lead to a gradual decay. Bottom (Scenario 3): In the intermediate arrhythmogenic regime (4%-18%), initial localized wave breaks evolve into persistent spiral waves. The sustained surge of the normalized wavefront length far beyond the baseline, accompanied by pronounced aperiodic oscillations, explicitly reflects the continuous generation, rotation, and chaotic meandering of self-sustaining ectopic wavefronts.

The distinction between these three scenarios is not based solely on visual inspection of **Figures 1**-**3**. To provide an objective characterization of the transition between stable propagation, transient fragmentation, and persistent spiral-wave activity, we introduced a quantitative analysis based on the normalized wavefront length, described in Section 3.1 and shown in **Figure 4**. This metric measures the total spatial extent of the active wavefront relative to the undisturbed planar wave before its interaction with the heterogeneous region. It therefore quantifies wavefront preservation, truncation, fragmentation, curling, and multiplication.

Using this quantitative measure, Scenario 1 is characterized by a transient reduction in wavefront length followed by rapid recovery to the planar-wave baseline. Scenario 2 is characterized by a sharp initial decrease, reflecting functional block and wavefront severing, followed by a moderate transient increase due to curling of the free wavefront ends and subsequent partial reattachment or decay. Scenario 3 is distinguished by a sustained increase far above baseline, accompanied by pronounced aperiodic oscillations and persistent wavefront tips, reflecting continuous generation and rotation of self-sustained spiral waves.

Together, these results demonstrate that dynamic cellular heterogeneity can induce distinct electrophysiological outcomes depending on the density of intermittently inactive cells: recovery of planar propagation at low density, transient wavefront fragmentation at high density, and sustained spiral-wave activity within an intermediate vulnerable range. Scenarios 2 and 3 therefore illustrate mechanisms by which intermittently inactive cells can promote pathological wave dynamics, including wave break, fragmentation, and reentry.

**Figure 5:**
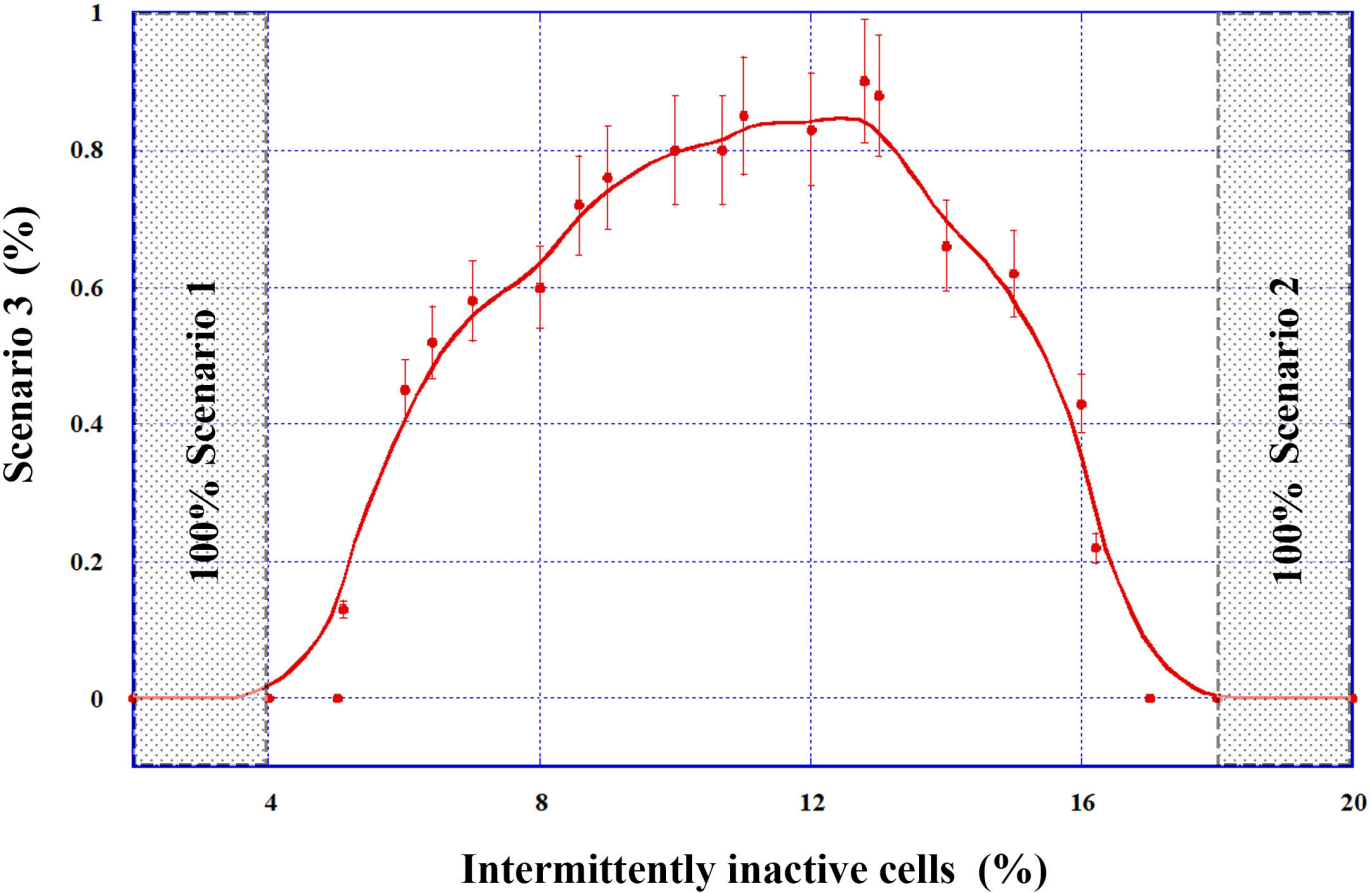
Parametric bifurcation map illustrating the probability of occurrence of the propagation regimes as a function of the density of intermittently inactive cells. The primary y-axis specifically plots the probability of emergence for Scenario 3 (persistent spiral waves). For each density value, 25 independent realizations were performed with different random spatial configurations of the intermittently inactive region. Each realization was classified into one of three regimes: uninterrupted planar propagation (Scenario 1), wavefront fragmentation (Scenario 2), or persistent spiral wave activity (Scenario 3) based on visual inspection alongside quantitative measures of normalized wavefront length (see Section 3.1). Data points represent the mean probability across the 25 realizations, and error bars indicate the standard error of the mean (SEM). Shaded regions denote the specific density domains where Scenarios 1 and 2 are dominant (occurring with 100% probability).

**Figure 5** illustrates a parametric map developed for the analyzed cardiac tissue region, aimed at assessing the electrophysiological behavior of intermittently inactive cells as a function of their spatial density. The primary purpose of this map is to identify critical concentration thresholds at which these intermittently inactive cells begin to significantly influence local excitability and, consequently, increase the risk of triggering arrhythmias. The map was generated through an extensive statistical analysis to robustly determine the probabilities of each regime. For every examined density value, 25 independent numerical realizations were performed, amounting to approximately 500 total simulations across the evaluated parameter space. Each realization utilized a newly randomized spatial distribution of the intermittently inactive cells within the defined rectangular region. Following the simulation, each realization was strictly classified into one of the three aforementioned propagation regimes. This classification was achieved through a combined approach: qualitative visual inspection of the spatiotemporal wavefront dynamics, corroborated by the objective quantitative metric of the Normalized Wavefront Length (as detailed in Section 3.1). The probability of occurrence for spiral wave activity (Scenario 3) was computed as the fraction of realizations exhibiting this behavior at a given density. Error estimates were calculated using the standard error of the mean (SEM) for each data point.

It should be emphasized that the critical thresholds delineating the transitions between these distinct regimes (at approximately 4% and 18%) were determined numerically rather than analytically. Because the system is governed by a stochastic temporal inactivation process, these transitions do not manifest as infinitely sharp phase boundaries. Instead, these values represent approximate density thresholds at which a clear shift in the dominant dynamical behavior is consistently observed. These numerically derived thresholds carry an inherent uncertainty on the order of a few percentage points due to the stochastic nature of the model and finite statistical sampling. Furthermore, it should be noted that these specific values (~4% and ~18%) are not universal physiological constants, but rather depend on the specific parameterization of our model. The precise location of these boundaries may shift under different conditions, such as alterations in the region’s geometry, the excitability parameter *a*, the diffusion coefficient, or the stimulation protocol. Nevertheless, the qualitative existence of the three distinct macroscopic propagation regimes remains fundamentally robust.

The results reveal three distinct behavioral regimes:

• When the proportion of intermittently inactive cells ranges between approximately 4% and 18% of the total cell population within the mapped region, the system operates in the dynamic regime associated with Scenario 3. In this intermediate range, the density of intermittently inactive cells is sufficient to markedly alter local conduction properties and facilitate the initiation of pathologic activity, as shown in **Figure 3**.
• At lower densities, specifically when the fraction of intermittently inactive cells falls below 4%, the tissue exhibits behavior consistent with Scenario 1. In this regime, the sparse presence of intermittently inactive cells is insufficient to overcome the protective mechanisms of the surrounding healthy myocardium, and the risk of pathological formations remains minimal, as depicted in **Figure 1**.
• Conversely, when the proportion of intermittently inactive cells exceeds 18%, the electrophysiological response corresponds to Scenario 2, as illustrated in **Figure 2**. In this case, the high concentration of inactive cells results in complete truncation of the advancing wavefront into two separate segments.

These findings indicate the presence of approximate, model-dependent transition densities that govern the shift from relatively benign (Scenario 1) to potentially pathological (Scenario 3) electrical behavior in the presence of intermittently inactive cells. Consequently, our results highlight the significant impact of passive cellular intermittency on wave propagation dynamics, demonstrating a spectrum of behaviors ranging from stable planar waves to fragmented wavefronts and, ultimately, to the emergence of persistent spiral waves. The ability of the wavefront to maintain functionality is contingent on the density and spatial distribution of inactive cells and other tissue properties, with these approximate boundaries delineating the transition between distinct propagation regimes.

**Figure 6:**
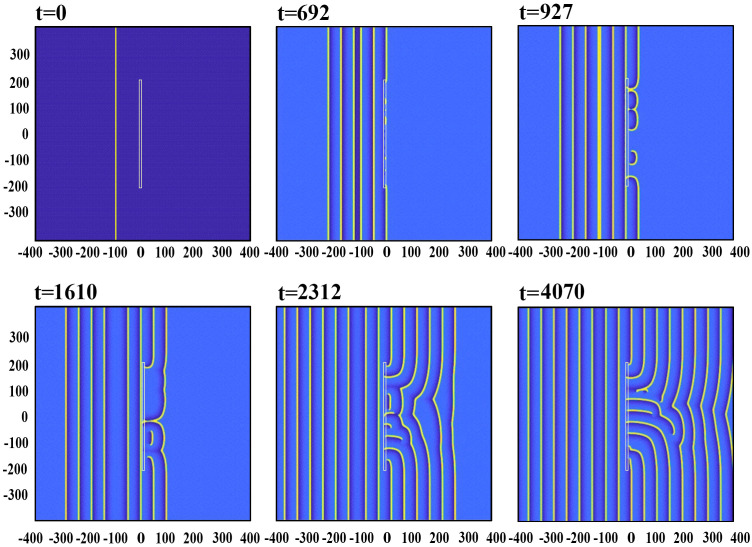
Autonomous spiral-wave activity under repeated planar-wave stimulation in Scenario 3 conditions. A train of incoming planar waves, applied at an inter-pulse interval (IPI) of 300 time units, interacts with the heterogeneous region (dashed rectangle: -7< X< 0 and |Y|< 200). Under this relatively high-frequency stimulation, spiral activity is repeatedly generated near the boundary of the intermittent region but remains largely confined to its vicinity, reflecting the influence of partial refractoriness in the surrounding tissue. See Supplementary Movie at https://physics.bgu.ac.il/~yaacov/File/serieswave.mp4 for the complete spatiotemporal evolution.

**Figure 7:**
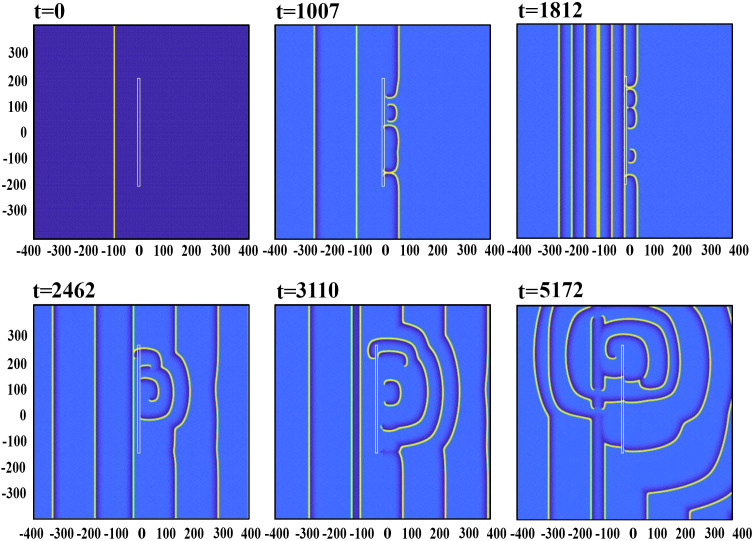
Autonomous spiral-wave activity under repeated planar-wave stimulation in Scenario 3 conditions. A train of incoming planar waves, applied at an inter-pulse interval (IPI) of 1000 time units, interacts with the heterogeneous region (dashed rectangle: -7< X< 0 and |Y|< 200). Under this lower-frequency stimulation, the surrounding tissue has sufficient time to recover excitability, allowing spiral waves to drift and progressively invade larger regions of the excitable medium. See Supplementary Movie at https://physics.bgu.ac.il/~yaacov/File/serieswave2.mp4 for the complete spatiotemporal evolution.

Physiological systems are rarely characterized by the propagation of a single wavefront. When the initial wave traverses the intermittently inactive region, our observations indicate that, under Scenario 3, it may give rise to additional ectopic sources that function independently. This raises an important question: what occurs when multiple waves propagate sequentially through the same region? To address this, we investigated the behavior of Scenario 3 under a series of planar waves. The resulting dynamics are presented in **Figures 6** and **7**. **Figure 6** depicts a condition in which the series of planar waves is applied at an inter-pulse interval (IPI) of 300 time units, whereas **Figure 7** illustrates a slower stimulation (IPI of 1000 time units).

The primary objective of these repeated-stimulation protocols is to phenomenologically demonstrate the frequency-dependent nature of wave break and spiral propagation. While the single-wave quantitative analysis (Section 3.1) provides objective evidence for persistent wavefront multiplication and free wave ends consistent with reentrant activity, **Figures 6** and **7** highlight the critical role of tissue restitution in modulating the spatial extent of spiral activity under repeated stimulation.

At higher stimulation frequencies (shorter inter-pulse interval, IPI = 300 time units, **Figure 6**), the rapid sequence of incoming wavefronts interacts with tissue that is still partially refractory. Consequently, the autonomous spiral wave activity generated by the intermittent zone remains heavily confined to the immediate boundary of the intermittent region. Crucially, while the intermittent region itself remains persistently active, functioning as a continuous, localized ectopic source; however, this spatial confinement prevents the spirals from invading the wider medium. As a result, the overall macroscopic state of the tissue is significantly less chaotic compared to the lower frequency regime. Conversely, at lower stimulation frequencies (IPI = 1000 time units, **Figure 7**), the surrounding healthy tissue is afforded sufficient time to fully repolarize and recover its excitability. Under these conditions, the development time of each ectopic source is prolonged, allowing the self-organized spiral waves to drift and progressively invade a much broader spatial domain of the surrounding tissue. This frequency-dependent spatial dispersion emphasizes the complex interplay between temporal intermittency and the fundamental restitution properties of the continuous excitable medium.

To assess whether the spiral waves are self-sustained, we performed additional simulations in which external periodic stimulation was discontinued after spiral formation. In these simulations, the spiral waves persisted and continued to rotate stably for the full remaining simulation time, corresponding to several hundred rotation periods. This behavior was reproducibly observed across independent realizations. These findings indicate that, in the intermediate-density regime, the dynamic heterogeneity introduced by intermittently inactive cells is sufficient to sustain spiral wave activity independently of continuous external stimulation.

**Figure 8:**
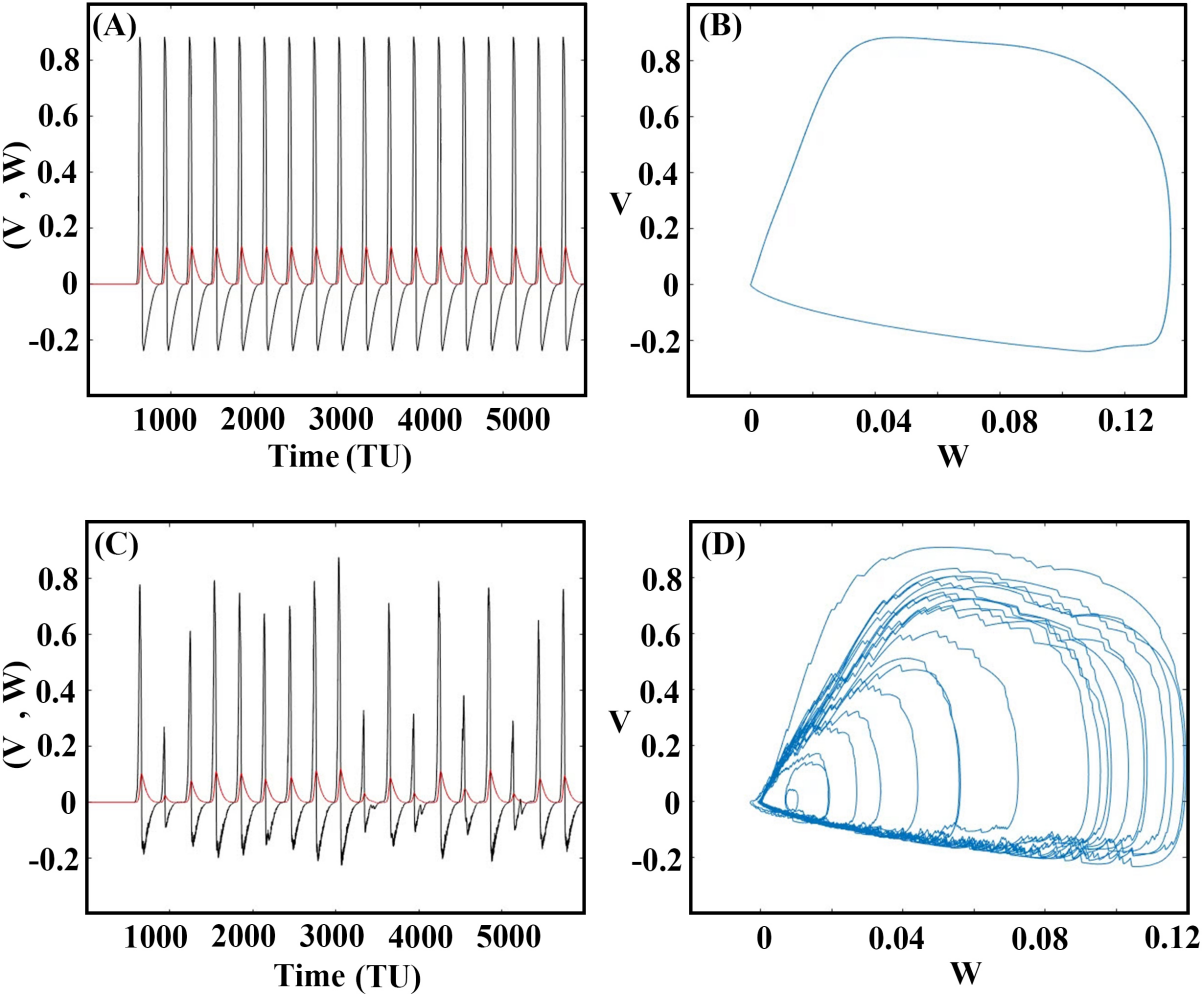
Temporal evolution and phase-space trajectories of the variables V (black) and W (red) at two representative spatial locations. **(A, B)** At a point distant from the heterogeneous region (X = -300, Y = 0), the system exhibits regular periodic oscillations and a well-defined limit cycle. **(C, D)** - At a point near the boundary of the intermittent region (X = 10, Y = 0), the dynamics display pronounced stochastic oscillations and a dispersed, chaotic phase space trajectory, indicative of a loss of dynamic stability and increased arrhythmogenic potential. The boundary of the intermittent region is defined by: -7<X<0 and |Y|<200.

**Figure 8** elucidates the temporal evolution and phase-space characteristics of the variables v and w, providing a detailed examination of wave propagation dynamics across heterogeneous cardiac tissue. Recall that *v* represents the transmembrane action potential in the FitzHugh-Nagumo (FHN) model. The analysis is conducted at two distinct spatial coordinates within the simulated two-dimensional grid, reflecting contrasting electrophysiological behaviors influenced by the presence of intermittently inactive cells. **Figures 8A, B** depict the temporal trajectory of *v* and its phase-space relationship with the inhibitory variable *w* at a reference point located in an unaffected region outside the intermittent zone, specifically at coordinates *X=-300*, *Y=0*. In contrast, **Figures 8C, D** illustrate the same metrics at a boundary-proximal location, *X=10*, *Y=0*, where the intermittent region interfaces with adjacent excitable tissue. The temporal evolution at the unaffected site (**Figures 8A, B**) exhibits a stable, periodic oscillation, consistent with the expected behavior of a uniformly excitable medium under controlled stimulation, reflecting the robust propagation of planar waves as observed in Scenario 1. Conversely, the boundary-proximal site (**Figures 8C, D**) reveals pronounced irregularities and stochastic fluctuations in *v*, characterized by abrupt transitions and variable amplitudes, indicative of dynamic disturbances arising from intermittent cellular activation. These fluctuations correlate with the wavefront fragmentation and spiral wave emergence observed in Scenarios 2 and 3, suggesting that the spatial gradient of excitability at the boundary amplifies the impact of stochastic inactivation (Rabinovitch et al., 2023). The phase-space plots further highlight these differences: the unaffected region displays a tightly constrained limit cycle, whereas the boundary region exhibits a dispersed trajectory, reflecting the loss of stability and the onset of arrhythmic potential. This comparative analysis underscores the critical role of spatial heterogeneity in modulating wave dynamics, providing a link between intermittent cell behavior and the initiation of complex electrical patterns in cardiac tissue.

### 3.1 Quantitative analysis of wave propagation using normalized wavefront length

#### 3.1.1 Rationale and definition of the metric

The representative simulations shown in **Figures 1**-**3** reveal qualitatively distinct patterns of excitation-wave propagation. However, visual inspection alone is insufficient for defining the boundaries between stable propagation, transient fragmentation, and sustained spiral-wave activity. To provide an objective and reproducible characterization of these regimes, we introduced a quantitative descriptor termed the Normalized Wavefront Length.

This metric was designed to quantify the instantaneous spatial extent of the active excitation wavefront during propagation. At each discrete time step, the total wavefront length was estimated from the set of excited elements forming the propagating activation front. The resulting instantaneous value was then normalized by the corresponding wavefront length of an undisturbed planar wave before interaction with the heterogeneous region, i.e., during the baseline interval t< 600 TU. Thus, a normalized wavefront length of approximately 1.0 represents a coherent planar wave propagating without substantial deformation.

Deviations from this baseline provide direct information about the structural state of the excitation wave. Values below 1.0 indicate shortening or truncation of the wavefront, typically associated with local conduction delay, partial block, or loss of excited segments. Values moderately above 1.0 indicate increased wavefront contour length caused by deformation, curling, or fragmentation. Sustained values far above 1.0, particularly when accompanied by irregular temporal oscillations, indicate continuous generation and rotation of additional wavefronts, as expected during persistent spiral-wave or reentrant activity.

In this sense, the normalized wavefront length provides an operational measure of propagation complexity. Rather than using a qualitative descriptor such as “more intricate,” the dynamics are quantified by the amplitude, persistence, and temporal variability of the normalized wavefront length. In particular, large excursions above the planar-wave baseline, together with aperiodic fluctuations and persistent wavefront tips, indicate a transition from transient wavefront deformation to sustained arrhythmogenic dynamics.

#### 3.1.2 Quantitative separation of the three propagation regimes

The temporal evolution of the normalized wavefront length clearly separated the three propagation regimes observed in the simulations (**Figure 4**).

In Scenario 1, corresponding to the low-density regime of intermittently inactive cells (< 4%), the planar wavefront was only transiently affected by the heterogeneous region. As the wavefront entered the region at approximately t = 600 TU, the normalized wavefront length decreased slightly, reaching a minimum value of about 0.8. This reduction reflects local wavefront truncation and small-scale conduction delays induced by sparse intermittently inactive cells. However, the perturbation was short-lived. By approximately t = 700 TU, the index returned to the baseline value of 1.0, indicating restoration of a coherent planar wavefront. The subsequent time course remained close to baseline, with only minor fluctuations. This behavior is consistent with wavefront recovery and uninterrupted macroscopic propagation.

In Scenario 2, corresponding to the high-density regime of intermittently inactive cells (> 18%), the heterogeneous region produced a much stronger disruption of the incoming planar wave. Upon entering the heterogeneous zone, the normalized wavefront length dropped sharply to approximately 0.4, indicating severe wavefront truncation and functional block. This abrupt decline corresponds to the severing of the original planar wavefront into disconnected propagating segments. The free ends of these segments subsequently curled and expanded, increasing the total active wavefront length. As a result, the normalized index rose above the planar-wave baseline and reached values of approximately 1.3. This increase reflects transient fragmentation and curling of the wavefront rather than sustained reentrant activity. At later times, the fragmented wave segments partially collided, reconnected, or decayed, producing a gradual reduction of the normalized wavefront length toward the baseline. Thus, Scenario 2 is characterized quantitatively by a sharp initial drop, a moderate transient overshoot, and a subsequent decay, consistent with strong wavefront fragmentation followed by partial functional recovery or termination of the fragmented pattern.

In Scenario 3, corresponding to the intermediate-density regime of intermittently inactive cells, approximately 4-18%, the normalized wavefront length displayed a qualitatively different temporal signature. The initial interaction with the heterogeneous region produced a modest decrease in the index, similar to the early response observed in Scenario 1. However, instead of returning to baseline, the system underwent a rapid and sustained increase in wavefront length. The normalized index rose far above 1.0 and reached values as high as approximately 5.0. This large increase indicates that the tissue no longer supported a single coherent propagating front; rather, multiple active wavefronts were continuously generated by rotating spiral activity.

The temporal profile in Scenario 3 also exhibited pronounced aperiodic oscillations. These fluctuations reflect the dynamic processes of spiral-wave rotation, meander, collision, annihilation, and regeneration of wavefront segments. Unlike Scenario 2, in which the increase in wavefront length was moderate and transient, Scenario 3 showed sustained elevation and irregular variability over time. This provides a quantitative signature of persistent ectopic activity and reentrant wave dynamics. Therefore, the intermediate-density regime represents a window of vulnerability in which the heterogeneous region is sufficiently dense to induce wave break, but not so dense as to completely suppress or terminate the resulting wavefronts. This balance enables the formation of self-sustained spiral waves.

#### 3.1.3 Interpretation of the regime boundaries

The normalized wavefront length analysis provides an objective basis for distinguishing the three regimes. In the low-density regime, the dominant quantitative feature is rapid recovery to the baseline value of 1.0 after a small transient reduction. In the high-density regime, the dominant feature is a sharp initial collapse of wavefront length followed by a limited transient overshoot and gradual decay. In the intermediate-density regime, the defining feature is a sustained and large-amplitude increase in wavefront length, accompanied by irregular temporal oscillations and persistent wavefront tips.

These quantitative differences support the classification of the propagation dynamics into three regimes: stable planar propagation, transient wavefront fragmentation, and persistent spiral-wave activity. The analysis also clarifies why the intermediate-density regime is the most arrhythmogenic among the three. Its increased complexity is not inferred solely from visual inspection, but is reflected in measurable properties of the wavefront dynamics: a substantially larger normalized wavefront length, prolonged persistence above baseline, and pronounced aperiodic temporal variability. These features indicate continuous generation and maintenance of active wavefronts, consistent with sustained reentry.

Thus, the normalized wavefront length serves as a compact quantitative measure linking the microscopic distribution of intermittently inactive cells to macroscopic wave dynamics. It demonstrates that dynamic cellular heterogeneity can produce distinct electrophysiological outcomes depending on density: recovery at low density, strong but transient fragmentation at high density, and persistent spiral-wave activity within an intermediate vulnerable range.

## 4 Discussion

In our recent study (Rabinovitch et al., 2023), we proposed, within a mechanistic modeling framework, that passive intermittent behavior of individual cardiomyocytes or localized groups of cardiomyocytes may constitute an important arrhythmogenic factor in atrial fibrillation (AF). In the present work, we extend that concept to a tissue-level continuous model in order to gain deeper insight into the spatiotemporal consequences of such intermittency. Whereas the previous study focused on varying the probability that a given cell would respond to an endogenous trigger, here we adopted a complementary approach: we fixed the probability of activation failure at 50% while varying the fraction of intermittently inactive cells within a predefined rectangular region. Although implemented differently, both formulations capture the same central concept of dynamic functional heterogeneity and allow us to examine how intermittent cellular behavior can destabilize wave propagation at the tissue scale.

The present simulations indicate that stochastic intermittency in cellular excitability can act as a potent and previously underexplored mechanism for disturbing wave propagation in otherwise structurally homogeneous tissue. In contrast to classical arrhythmogenic substrates based on fixed anatomical heterogeneities such as fibrosis, scar formation, or permanent conduction barriers, our results show that temporally fluctuating excitability alone may be sufficient to induce substantial conduction disturbances. In this sense, the model highlights the possibility that functional heterogeneity, even in the absence of permanent structural defects, can initiate and sustain complex propagation patterns, including wave break and reentrant activity.

A central finding of this study is the density-dependent transition between distinct propagation regimes. At low levels of intermittency, the wavefront remains largely intact, exhibiting only minor local distortions and preserving global propagation. At intermediate densities, however, the heterogeneous region becomes sufficiently disruptive to generate local wave breaks that can evolve into sustained spiral activity. At still higher densities, conduction through the affected region becomes severely compromised, leading primarily to fragmentation and failure of coherent transmission. This nonmonotonic behavior is particularly noteworthy, as it suggests that the most arrhythmogenic regime is not necessarily the one with the greatest level of inactivation, but rather an intermediate state in which conduction remains possible yet becomes highly unstable. Such a regime provides favorable conditions for the initiation of self-sustained reentrant dynamics.

In light of these dynamics, it is important to explicitly distinguish the role of temporal intermittency from purely spatial heterogeneity. While our baseline conditions establish the stability of a pristine homogeneous medium (prior to the wavefront entering the intermittent zone), the specific non-monotonic emergence of spiral waves strongly points to the dynamic nature of the substrate as the primary arrhythmogenic driver. Sustained spiral wave activity emerged exclusively within the intermediate range of inactivation density (4%-18%), rather than at higher densities (>18%) which inherently present a larger degree of spatial structural disruption. If static spatial heterogeneity were the sole driver, conduction disruption would be expected to increase monotonically with obstacle density. Furthermore, the ability of these localized fragments to evolve into persistent, self-sustaining spiral waves long after the initial planar wavefront has passed implies that the ongoing, stochastic state-switching of the cells actively feeds and maintains the reentrant circuits. These dynamical signatures indicate that the time-varying nature of cellular intermittency appears to play an active role in generating the complex electrical instability observed, well beyond the passive wave-breaking expected from static anatomical obstacles alone.

From a physiological perspective, these findings support the idea that intermittent loss of excitability may represent a plausible functional contributor to AF initiation and maintenance. Although the present model is intentionally simplified and does not attempt to reproduce the full electrophysiological complexity of atrial tissue, it captures an important mechanistic principle: dynamic, localized fluctuations in excitability can create a substrate in which orderly wave propagation becomes vulnerable to breakup, local source formation, and persistent rotational activity. In this respect, the study offers a possible bridge between cellular-scale intermittency and tissue-scale arrhythmogenic behavior.

An additional conceptual parallel may be drawn from the literature on intermittent faults (IFs) in electronic systems. Such faults are known to appear, disappear, and recur unpredictably, often posing major diagnostic and operational challenges (Rashid et al., 2015). In many cases, intermittent faults become more persistent over time and may eventually evolve into permanent failures (Correcher et al., 2012). Their localization in complex electronic systems is also notoriously difficult, and a broad range of diagnostic approaches has been developed to address this problem (Zhou et al., 2020). While the biological and electronic contexts are clearly different, this analogy is nevertheless useful: in both systems, transient and spatially localized failures can have disproportionately large effects on global system behavior. In the cardiac setting, such intermittency may similarly evade straightforward detection while still exerting a profound influence on wave dynamics and electrical stability.

### 4.1 Robustness to background noise and threshold dynamics

To assess the robustness of the observed propagation regimes to stochastic perturbations, we performed additional simulations in which additive Gaussian background noise was introduced into the fast excitation variable v of the FitzHugh-Nagumo equations. The maximum noise amplitude was set to 0.01. This value was chosen deliberately as a weak-noise perturbation, small compared with the characteristic excursion of the excitation variable during a propagating action-potential-like wave, where v reaches values of order unity. The purpose of this test was therefore not to map the full response of the system to noise intensity, but rather to examine whether the three density-dependent regimes identified in the intermittent-switching framework remain stable under low-amplitude stochastic fluctuations.

Under this weak-noise perturbation, the three distinct propagation regimes remained qualitatively unchanged. At low densities of intermittently inactive cells, the planar wavefront continued to propagate through the heterogeneous region with only minor distortions and recovered its global coherence. At high densities, the heterogeneous region still produced strong wavefront disruption and fragmentation. Most importantly, in the intermediate-density regime, persistent spiral-wave activity continued to emerge and was not suppressed by the added background noise. Thus, weak Gaussian noise neither generated a new dominant macroscopic regime nor eliminated the density-dependent transition from stable propagation to fragmentation or sustained spiral-wave dynamics. Representative noisy simulations for the three propagation scenarios are provided in **Supplementary Material 1**. In these simulations, all model parameters, boundary conditions, stimulation protocols, spatial discretization, and numerical integration settings were kept identical to those used in the corresponding noiseless simulations described in the main text; the only modification was the addition of weak additive Gaussian background noise to the fast excitation variable v. The movies illustrate the low-density, intermediate-density, and high-density cases under the same weak-noise perturbation used in the robustness analysis. They confirm that this perturbation does not qualitatively alter the macroscopic propagation patterns: coherent planar propagation is preserved at low density, persistent spiral-wave activity remains present at intermediate density, and wavefront fragmentation remains the dominant response at high density.

The limited effect of this weak stochastic forcing can be understood from the excitability structure of the FitzHugh-Nagumo model. In regions occupied by functionally inactive cells, corresponding to a = 0.54 in the present parameterization, small fluctuations in v typically decay without triggering a regenerative excitation. Similarly, when noise is superimposed on the plateau of an already developed excitation wave, where v is close to its activated state, it does not substantially alter the macroscopic wave morphology. Noise can affect propagation most efficiently only when a local region of the medium is positioned close to the effective excitation threshold. In our parameterization, this threshold is approximately v ≈ 0.185. Because the imposed noise amplitude is small relative to the distance between the resting state and this activation threshold, noise-induced threshold crossings are expected to be rare. Consequently, the dominant source of variability in the present simulations remains the imposed stochastic switching of intermittently inactive cells, rather than the added background noise.

It is important, however, to distinguish this weak-noise robustness test from a systematic investigation of noise-driven pattern formation. Additive noise in spatially extended excitable media is known to have strongly amplitude-dependent effects. In particular, Perc (2005) studied a two-dimensional FitzHugh-Nagumo excitable medium with additive Gaussian noise and demonstrated spatial coherence resonance, showing that an intermediate level of noise can optimally enhance an intrinsic spatial scale of the medium. The same study also showed that at sufficiently high noise levels, random fluctuations may dominate the spatial dynamics and reduce coherent structure.

Accordingly, the present noise analysis should be interpreted as evidence that the three regimes reported here are robust to the weak stochastic perturbation tested here, rather than as a complete characterization of all possible noise amplitudes. Although no qualitative changes were observed at this noise level, stronger noise amplitudes may constitute an additional dynamical control parameter in FitzHugh-Nagumo excitable media and could potentially interact with the intermittent-inactivation mechanism. A systematic exploration of such stronger-noise regimes therefore remains an interesting direction for future work.

Taken together, these results reinforce the view that passive intermittent cellular behavior deserves further attention as a potential source of arrhythmogenic instability. More broadly, they suggest that dynamic functional heterogeneity should be considered alongside structural remodeling in efforts to understand mechanisms underlying atrial fibrillation. The importance of local heterogeneity and delayed activation in shaping collective dynamics has also been emphasized in other biological excitable tissues. For example, Stožer et al. (2019) highlighted heterogeneity and delayed activation as hallmarks of self-organization and criticality in pancreatic islets. Although the physiological context differs from cardiac tissue, their findings support the broader theoretical principle that spatial and temporal variability in cellular excitability can have profound macroscopic consequences for tissue-level dynamics. While the qualitative emergence of the three regimes is robust in the present model configuration, the exact transition densities identified here, approximately 4% and 18%, are inherently tied to the specific model parameterization, geometry, and stimulation protocol used in this study. In particular, these boundaries are expected to depend not only on the shape, but also on the spatial extent and aspect ratio of the intermittently inactive region, since these factors determine both the number of intermittently inactive cells encountered by the propagating wavefront and the effective distance over which the wavefront interacts with the heterogeneous substrate. Future work should therefore examine how variations in the dimensions of the intermittently inactive region, together with anisotropic conduction, realistic ionic kinetics, anatomically structured tissue, background noise, inter-pulse interval, and topological diagnostics of spiral-wave activity, influence the location of these regime boundaries.

## 5 Conclusions

This study identifies intermittent cellular inactivation as a distinct form of dynamic functional heterogeneity capable of profoundly altering wave propagation in excitable cardiac-like media. Within the framework of our continuous two-dimensional FitzHugh-Nagumo model, we show that even in the absence of fixed anatomical obstacles, localized stochastic fluctuations in excitability can destabilize an otherwise ordered propagating wave and give rise to qualitatively different electrophysiological regimes. In this sense, the present results extend the concept of arrhythmogenic substrates beyond purely structural abnormalities and emphasize the potential importance of transient, functionally mediated conduction disturbances.

A central outcome of the study is the identification of threshold-dependent transitions in propagation behavior. At low densities of intermittently inactive cells, wave propagation remains largely preserved, with only minor local perturbations. At intermediate densities, however, the system enters a particularly vulnerable regime in which wave breaks can evolve into persistent spiral activity. At higher densities, conduction through the affected region becomes severely impaired, resulting primarily in wavefront fragmentation and loss of coherent transmission. Quantitatively, these transitions occur in our model around density levels of approximately 4% and 18%, thereby defining a nontrivial window in which dynamic instability is maximized.

These findings suggest that intermittency in cellular excitability may represent a plausible mechanistic contributor to the initiation and maintenance of arrhythmia-like behavior, particularly under conditions in which tissue structure remains macroscopically intact. More broadly, the results support the view that excitable-media instability can emerge not only from permanent anatomical remodeling, but also from localized stochastic fluctuations in cellular responsiveness. This perspective may be especially relevant for understanding situations in which clinically significant electrical dysfunction arises in tissue that does not exhibit overt structural damage.

From a translational standpoint, the present work highlights the importance of considering dynamic excitability heterogeneity in future computational and conceptual models of atrial fibrillation. Although the current study is based on a reduced phenomenological model and therefore does not attempt to reproduce the full physiological complexity of atrial tissue, it provides a useful mechanistic framework for investigating how transient local conduction failure may initiate larger-scale electrical instability. Future studies should test the robustness of this mechanism in more detailed ionic and anatomically realistic models, and should further explore whether comparable signatures of intermittent excitability can be linked to experimentally or clinically observed patterns of conduction instability.

Taken together, our results reinforce the idea that passive intermittent cellular behavior deserves further consideration as a potential arrhythmogenic substrate. By demonstrating that stochastic local inactivation alone can drive the transition from stable propagation to spiral-wave dynamics, this study contributes to a broader understanding of how dynamic functional disturbances may participate in the emergence of complex cardiac rhythm disorders.

## Data Availability

The original contributions presented in the study are included in the article/**Supplementary Material**. Further inquiries can be directed to the corresponding author.

## Supplementary material

Supplementary Movie S1 Scenario 1 under weak background noise. Low-density case showing preserved planar-wave propagation. https://physics.bgu.ac.il/~yaacov/File/Scenario1noise001.mp4.

Supplementary Movie S2 Scenario 2 under weak background noise. High-density case showing persistent wavefront fragmentation. https://physics.bgu.ac.il/~yaacov/File/Scenario2noise001.mp4.

Supplementary Movie S3 Scenario 3 under weak background noise. Intermediate-density case showing persistent spiral-wave activity. https://physics.bgu.ac.il/~yaacov/File/Scenario3noise001.mp4.
